# Ultrasound-guided Erector Spinae Plane Block: Indications, Complications, and Effects on Acute and Chronic Pain Based on a Single-center Experience

**DOI:** 10.7759/cureus.3815

**Published:** 2019-01-02

**Authors:** Serkan Tulgar, Onur Selvi, Ozgur Senturk, Talat E Serifsoy, David T Thomas

**Affiliations:** 1 Anaesthesiology, Maltepe University Faculty of Medicine, Istanbul, TUR; 2 Aneasthesiology, Maltepe University Faculty of Medicine, Istanbul, TUR; 3 Anesthesiology, Maltepe University Faculty of Medicine, Istanbul, TUR; 4 Anesthesiology, Maltepe University Faculity of Medicine, Istanbul, TUR; 5 Pediatric Surgery, Maltepe University Faculty of Medicine, Istanbul, TUR

**Keywords:** regional anesthesia, pain, erector spinae block, espb, erector spinae plane block

## Abstract

Introduction: Erector spinae plane block (ESPB) is a novel regional anesthesia technique used in postoperative pain and chronic neuropathic pain of the thoracoabdominal region. There are no previously published large case series. This retrospective review aimed to report the indications, levels of block, success of block and complications, and also to evaluate the effect of ESPB on postoperative/chronic pain.

Methods: We retrospectively evaluated the charts and medical records of 182 patients who had ESPB in the last one year. All records were collected in the postoperative recovery room, ward, and pain unit.

Results: ESPB performed at several different levels and for several different indications led to effective postoperative analgesia when part of a multimodal analgesia plan. Few complications were noted.

Conclusion: ESPB is an interfascial plane block with many indications. The possibility of complications must be considered.

## Introduction

Ultrasound-guided erector spinae plane block (ESPB) is an interfascial plane block described by Forero et al. [1] for the treatment of thoracic neuropathic pain. Although first described for use in chronic pain, it has later been used as a postoperative analgesia method in many surgical procedures from shoulder to hip surgeries [2,3,4]. There are only a limited number of randomized clinical trials of ESPB [5,6,7] and only a few studies have reported complications [8,9]. In a recently published pooled review, 85 ESPB-related publications published in 21 journals were analyzed [10]. A single centre experience of this intriguing technique has yet to be published.

Anatomical dissections and imaging studies aimed at revealing the mechanism of the effect of ESPB have reported differing results [11,12,13]. Technique and level of application, concentration and volume of the local anaesthetic (LA), descriptive features of the patients, and several other factors affect the success rate of ESPB and its coverage area [14,15]. Therefore the operator and/or technique may play an important role in the block’s success.

In this retrospective review, we report the indications, levels of block, success of block, and effect of ESPB on postoperative analgesic effect.

## Materials and methods

Study design

After local ethical committee approval and registration at clinicaltrials.gov (NCT03739086), a patient chart review was performed. Prospectively collected data were retrospectively analyzed. Patients undergoing ESPB in our center between 01.07.2017 and 01.10.2018 were included in the study. Patients who underwent a peripheral block in addition to ESPB were not included in the study.

Data collection

A standard peroperative and postoperative analgesia plan is applied to all patients who undergo a regional anesthesia technique at our institute and a standardized regional anesthesia data collection form is used to collect all patient data. All patients undergoing regional anesthesia techniques give informed consent for all procedures and the use of their data in medical studies.

The following data were collected for all patients undergoing ESPB: age, gender, weight, height, surgical procedure, surgical time, patient-controlled analgesia (PCA) (tramadol) data, use of routine and rescue analgesia, and numeric rating scale (NRS) at 1st, 3rd, 6th, 9th, 12th, 18th, and 24th hours.

Additionally, descriptive data were noted from data collection forms. In patients undergoing ESPB, the level of ESPB, unilateral or bilateral application, volume applied, concentration and LA content, time of block (preoperatively under sedoanalgesia, following induction, or at end of surgery under general anesthesia), block application time, complications, and any additional descriptive data the patient provided during follow-up was noted. While there is no standard bupivacaine concentration for our block application, in patients undergoing ESPB for short-lasting surgical procedures (less than one hour) or ESPB after completion of surgery, the LA included 0.4%-0.5% lidocaine in addition to bupivacaine. Maximum of 150 mg or 2.5 mg/kg bupivacaine and maximum 200 mg or 3 mg/kg of lidocaine was used. ESPB was performed under ultrasound guidance and generally using the out-of-plane technique.

Standard perioperative analgesia included paracetamol 1 gr and tenoxicam 20 mg. However, in patients undergoing major surgical procedures such as thoracotomy, laparoscopic hysterectomy, hip surgery or laparoscopic/open nephrectomy, 0.05 mg/kg morphine (max 3.5 mg) was added. Our postoperative analgesia plan included paracetamol application every eight hours as standard. However, if the NRS value was <2 and the patient did not request analgesia, paracetamol doses were skipped or delayed. Additionally, tramadol PCA (basal infusion free, 10 mg bolus, 20 min lockout) was also standard and commenced in the recovery room. If despite these analgesics the NRS value was >3, rescue analgesia was performed using intramuscular diclofenac and, if required, meperidine 50 mg. All analgesics and their application times were noted in detail in patient files.

Patients with NRS values ≥6 within the first hour or those requiring rescue analgesia within the first six hours were considered as block failure/lack of efficiency. Also, patients with an NRS value <4 within the first 24 hours who do not meet the criteria for block failure or who reported reflected pain were considered as “inadequate spread for surgical procedure”.

In addition to data of the patients undergoing ESPB for postoperative pain control, data of the patients undergoing ESPB for chronic pain were also obtained from the patient files.

## Results

The data of 182 patients undergoing ESPB between 01.07.2017 and 01.10.2018 were included in the study. ESPB was performed for postoperative analgesia in 173 patients and chronic pain in nine patients. The average age of the patients was 58.8 years (range 8 y-88 y) and the average body mass index was 27.9 (16.2-42.9). The American Society of Anesthesiology (ASA) physical status rating of patients undergoing ESPB for postoperative analgesia were '1' for 41 patients, '2' for 101 patients, and '3' for 31 patients.

We found that 15-30 ml of local anaesthetic was used per side and level. The maximum applied LA volume was 60 mL. While 88 patients underwent bilateral ESPB, 94 underwent unilateral ESPB. When considering bilateral and bi-level blocks, a total of 296 ESPB applications were performed.

ESPB was performed using the in-plane technique in only 14 patients; the remaining underwent ESPB using the out-of-plane technique. In a majority of patients, lidocaine was added to the LA and bupivacaine was used in 0.25% concentration.

ESPB for postoperative analgesia

Block application levels, local anaesthetic concentrations and applied volumes, surgical procedures, average paracetamol use, and tramadol requirements in the first 24 hours as well as the first 24-hour average NRS scores are shown in Tables 1-5. ESPB was performed preoperatively under sedoanalgesia in 40 patients, after anaesthesia induction and before surgical procedure in 69, under general anaesthesia after completion of surgical procedure in 63, and under spinal anaesthesia after completion of surgical procedure in one patient. In patients undergoing shoulder arthroscopy, breast surgery, and thoracotomy, single level ESPB between Th2-Th5 was used in 32 patients and bi-level ESPB between Th4-Th6 was performed in 11 patients.

**Table 1:** Levels, local anaesthetic concentration, analgesic agent requirement and average NRS scores in patients undergoing high thoracic ESPB. NRS - numeric rating scale, ESPB - erector spinae plane block, Th: Thoracic

	Bupivacaine %0.25/%0.375 (n)	Paracetamol (gr/day)	Tramadol (mg/day)	Average NRS (min-max)	No of Patients Requiring Rescue Analgesia
Th2 (n:5)					
Shoulder arthroscopy (20 mL)	5/0	2,20 (2-3)	112 (40-280)	2,25 (0-8)	2
Th3 (n:1)					
Shoulder arthroscopy (20 mL)	0/1	2 (1-3)	80	2 (1-4)	0
Th5 (n:26)					
Mastectomy (20 ml)	5/2	1.50 (0-3)	62.5 (20-100)	1,55 (0-4)	0
Mastectomy+axillary dissection (30 mL)	7/0	1.85 (0-3)	23.5(0-90)	1,36(0-6)	1
Mastectomy+prosthesis+abdominoplasty (bilateral,30+30mL)	1/0	2	0	1,25 (0-3)	0
Mastectomy+thoracic wall revision (30 mL)	0/1	2	100	1.25 (0-3)	0
Thoracotomy (30 ml)	8/2	3	151 (60-240)	2,28 (0-7)	5
Th4-Th6 bi-level (n:11)					
Thoracotomy (15+15 mL)	9/2	2.63 (1-3)	90 (0-280)	1,74 (0-5)	2

**Table 2:** Levels, local anaesthetic concentration, analgesic agent requirement and average NRS scores in patients undergoing Th6-Th9 ESPB. NRS - numeric rating scale, ESPB - erector spinae plane block, Lap - laparoscopic, ERCP - endoscopic retrograde cholangiopancreatography

	Bupivacaine %0.25/%0.375 (n)	Paracetamol (gr/day)	Tramadol (mg/day)	Average NRS (min-max)	No of Patients Requiring Rescue Analgesia
Th6 (n:9)					
Sleeve gastrectomy (bilateral, 30 mL-30 mL)	2/0	3	150 (0-300)	2.5 (1-8)	1
Total gastrectomy (bilateral, 30 mL-30 mL)	1/0	2	100	1,75 (1-3)	0
Lap. Nissen fundoplication (bilateral, 30 mL-30 mL)	4/2	2,66 (2-3)	180 )120-280)	2,13 (1-8)	4
Th8 (n:8)					
Lap. Nissen fundoplication (bilateral, 30 mL-30 mL)	3/1	1,5 (1-3)	115 (60-200)	1,44 (0-4)	1
Incisional hernia after open cholecystectomy (20 mL)	1/0	1	0	1 (0-3)	0
Lap. umbilical hernia (20 mL)	0/2	1,5 (1-2)	25 (0-50)	1,38 (1-4)	0
Lap. cholecystectomy+ERCP (30 mL)	1/0	1	130	2 (0-3)	0
Th9 (n:51)					
Lap. cholecystectomy (bilateral, 20 mL-20 mL)	26/15	2,31 (1-3)	107,31 (40-270)	1,75 (0-6)	7
Laparoscopic nephrectomy (bilateral, 20 mL-20 mL)	1/0	2	40	1,50 (0-4)	0
Open nephrectomy (20 mL)	1/0	3	110	1,75 (1-4)	0
Open bladder surgery (bilateral, 30 mL-30 mL)	0/1	2	40	1,5 (1-3)	0
Lap. hysterectomy (bilateral, 20 mL-20mL)	1/0	2	0	2 (1-4)	0
Lap. inguinal hernia repair (bilateral, 20 mL-20mL)	1/0	3	0	1,75 (0-3)	0
Open retropubic prostatectomy (bilateral, 20 mL-20mL)	0/5	1,6 (0-3)	82 (40-200)	1,45 (0-4)	0

In patients undergoing laparoscopic/open upper or lower abdominal, urological or gynaecological procedures, 68 underwent single level ESPB between Th6-Th9 and two underwent bi-level ESPB between Th9-L2 (thoracolumbar) levels. ESPB was performed from the low thoracic vertebral levels (Th10-11) in 17 patients undergoing abdominal surgeries. ESPB was performed from the lumbar vertebral levels in 43 patients undergoing urological, gynaecological, hip, and knee surgeries. The details of surgical procedures and descriptive data are given in Tables 1-4.

**Table 3:** Levels, local anaesthetic concentration, analgesic agent requirement and average NRS scores in patients undergoing lower thoracic ESPB. *: in this patient, motor weakness was observed) NRS - numeric rating scale, ESPB - erector spinae plane block, Lap - laparoscopic

	Bupivacaine %0.25/%0.375 (n)	Paracetamol (gr/day)	Tramadol (mg/day)	Average NRS (min-max)	No of Patients Requiring Rescue Analgesia
Th10 (n:11)					
Open inguinal hernia repair (unilateral, 30 mL)	3/0	1,66 (1-2)	106 (80-130)	1,75 (0-4)	0
Open inguinal hernia (bilateral, 30 mL-30 mL)	1/0	3	180	3,25 (2-7)	1
Right hemicolectomy (bilateral, 20mL-20 mL)	1/0	2	60	2,50 (0-4)	0
Lap. Nissen fundoplication (bilateral, 30 mL-30 mL)	1/0	3	140	1,25 (0-3)	0
Lap. adrenalectomy (bilateral, 30 mL-30 mL)	0/1	2	0	2 (1-3)	0
Lap. hysterectomy (bilateral; 20 mL-20 mL)	3/0	2,33 (1-3)	180 (30-280)	3,08 (0-8)	2
Lap. ovarian cystectomy (bilateral 20 mL-20 mL)	1/0	3	80	1,5 (0-3)	0
Th11 (n:6)					
Ileus-Tm. resection (bilateral, 20mL-20 mL)	1/0	3	80	1,50 (0-3)	0
Lap. hysterectomy (bilateral; 20 mL-20 mL)	1/0	2	140	1,50 (0-3)	0
Lap. ovarian cystectomy (bilateral 20 mL-20 mL)	2/0	2 (1-3)	140	1,38 (0-4)	0
Cesarean section (bilateral 25 mL-25 mL*)	1/0	3	40	1,5 (0-3)	0
Radical prostatectomy (bilateral 30 mL-30 mL)	1/0	3	150	1,75 (0-4)	1
Th9-L2 Bilevel (n:2)					
Incisional hernia after open nephrectomy, flank (bilevel, unilateral; 20 mL-20 mL)	1/0	3	110	1,5 (0-3)	0
Lap. hemicolectomy (bilevel, bilateral; 15/15/15/15 mL)	1/0	2	0	1,25 (0-4)	0

When postoperative 24-hour analgesia requirements were analyzed, 41 patients (23%) required rescue analgesia, 15 (9%) of whom required rescue within the first 12 hours. The average paracetamol and tramadol use per patient was 2.33 gr (0-3 gr) and 99.33 (0-300) mg, respectively. The average NRS score in the first 24 hours was 1.86 (average range for all patients 0-4.75). The analgesic use, rescue analgesia requirement, and NRS score distributions are shown in Tables 1-4.

**Table 4:** Levels, local anaesthetic concentration, analgesic agent requirement and average NRS scores in patients undergoing lumbar ESPB. (*: This patient underwent lumbar ESPB from L4 with only 30 mL of local anaesthetic due to the patient’s short stature, **: Only 18 mL of local anaesthetic was applied in this pediatric patient (0.5 mL/kg).) NRS - numeric rating scale, ESPB - erector spinae plane block

	Bupivacaine %0.25/%0.375 (n)	Paracetamol (gr/day)	Tramadol (mg/day)	Average NRS (min-max)	No of Patients Requiring Rescue Analgesia
L1 (n:2)					
Pyeloplasty (bilateral; 20 mL-20 mL)	1/0	1	30	1 (0-2)	0
Radical prostatectomy (bilateral; 20 mL- 20 mL)	1/0	1	0	1,25 (0-3)	0
L4 (n:41)					
Total hip replacement (30 mL)*	1/0	1	40	1 (1-3)	0
Total hip replacement (40 mL)	11	2,90 (2-3)	130 (40-220)	2,44 (1-8)	4
Partial hip replacement (unilateral, 18 mL**)	15	2,66 (2-3)	120 (60-220)	2,10 (0-7)	5
Proximal femur nail (40 mL)	7/0	2 (1-3)	104 (50-240)	2,14 (0-6)	3
Femur lengthening (40 mL)	2/0	2,5 (2-3)	100 (80-120)	1,5 (0-4)	0
Thigh Tm. (40 mL)	1/0	1	130	1,44 (0-5)	1
Knee prosthesis removal & spacer (40 mL)	1/0	2	140	1 (0-3)	0
Femur shaft fracture (40 mL)	3/0	2,33 (2-3)	70 (0-140)	1,5 (0-6)	1

ESPB for chronic pain

Five patients underwent ESPB from Th2-Th3 for frozen shoulder, three from Th4 for myofascial pain, and one patient from L4 for postoperative pain. The average NRS scores were 7.11 (range 5-8) before ESPB, and following ESPB, the average NRS decreased to 1.22 (0-2) at the 1st hour, 1.44 (0-3) at the 24th hour, and 2 (0-3) at the 72nd hour. The patients reported a high degree of satisfaction on the third day. ESPB application details are given in Table 5.

**Table 5:** ESPB results in patients undergoing ESPB for chronic pain. NRS - numeric rating scale, ESPB - erector spinae plane block

	NRS Before ESPB	NRS 1 hour after ESPB	NRS 24 hours after ESPB	NRS 72 hours after ESPB	Change in Degree of Abduction Degree (Before ESPB and 1 hour after)
Th2 (n:5)					
Frozen shoulder (20 mL)	8	2	2	3	30°-135°
Frozen shoulder (20 mL)	8	2	2	2	45°-135°
Frozen shoulder (20 mL)	7	1	1	2	30°-120°
Frozen shoulder (20 mL)	8	2	3	3	45°-135°
Frozen shoulder (20 mL)	8	2	2	3	45°-135°
Th4 (n:3)					
Lower cervical and interscapular myofascial pain (bilateral; 20 mL-20 mL)	6	0	0	0	
Lower cervical and interscapular myofascial pain (bilateral; 20 mL-20 mL)	6	0	1	1	
Interscapular myofascial pain (bilateral; 15 mL-15 mL)	5	0	0	1	
L4 (n:1)					
Neuropathic pain after hip surgery (40 mL)	8	2	2	3	

Block failure/lack of efficiency and inadequate spread for surgical procedure

ESPB failure/lack of efficiency was seen in 12 patients (6.5%). There was no common denominator of these patients with regards to surgical procedures, application level, applied LA volume or concentration. Surgical procedures in these patients were thoracotomy in three, laparoscopic hysterectomy in three, hip surgery in two, and sleeve gastrectomy, shoulder arthroscopy, laparoscopic Nissen fundoplication, and open inguinal hernia repair in one patient each. Prolonged surgery time for thoracotomies may have led to a decrease in or loss of ESPB effect.

In 10 patients (5.5%) ESPB provided effective analgesia but patients reported mild to moderate pain in a section of the surgical field or reflected pain and therefore the block was considered to be “inadequate”. While two patients undergoing mastectomy and axillary dissection did not report breast pain, they reported mild to moderate pain over the axilla. In seven patients undergoing Nissen fundoplication, all seven reported left shoulder pain but no pain in the surgical field. One patient undergoing Nissen fundoplication reported both shoulder pain and mild to moderate mid abdominal pain.

Complications

Complications were seen in four (0.22%) patients. In a 29-year-old female undergoing C/S and myomectomy, bilateral ESPB (15 ml 0.5% bupivacaine, 5 ml 2% lidocaine and 5 ml saline solution for a total volume of 25 ml per side) was performed from Th11 following completion of surgery. In this patient bilateral quadriceps muscle weakness was observed for 14 hours. The patient had no previous history of neurological disease. 

Bilateral ESPB was performed from Th9 under general anaesthesia following completion of surgery in a 56-year-old female patient who underwent laparoscopic hysterectomy. Transient apathy and aphasia was observed for three hours, possibly due to ESPB or the effects of general anesthesia.

A 38-year-old female underwent ESPB from Th3 for myofascial pain. Following ESPB, perioral numbness, lisp, and dizziness were observed. These symptoms fluctuated until complete resolution after 1.5 hours. This was probably a vascular complication of lidocaine. 

A 76-year-old female patient who underwent laparoscopic umbilical hernia repair underwent bilateral ESPB from Th9 preoperatively without sedation. Following ESPB, the patient was observed to have loss of general muscle tonus and consciousness. Following airway control and O2 support, the patient was unconscious until the third minute and the Glasgow coma scale (GCS) was 15 after 10 minutes, after which the surgery was successfully completed. We believe this is possibly a minor neurological complication of the LA. No treatment was required.

These complications were considered to be due to the spread of the LA to the lumbar plexus in the first case and probable LA toxicity in the remaining cases. In the second patient, atropine-related anticholinergic syndrome could not be ruled out.

## Discussion

Herein we reported a total of 182 patients undergoing ESPB from 13 different level/combinations of which nine were thoracic, two were separate lumbar, and two were bilevel. While most ESPBs were performed for postoperative analgesia, we also report a limited number of patients undergoing ESPB for chronic pain.

The use of ESPB for postoperative analgesia has increased in popularity with new indications continuing to be reported [5,6,10,16]. In addition to previously published indications, we report the use of ESPB in previously unreported surgeries such as laparoscopic/open renal and perirenal procedures and procedures requiring large dermatomal blockage such as mastectomy+breast prosthesis+abdominoplasty, urological procedures such as bladder surgeries, pyeloplasty and radical prostatectomy, gynecological procedures, and orthopedic procedures such as knee and thigh surgeries.

While ESPB has been reported for use in frozen shoulder and myofascial pain, we report its first time use from L4 in neuropathic pain following hip surgery. ESPB was performed in 34 different surgical procedures and three different causes of chronic pain.

We report ESPB performed from different levels or a combination of levels depending on the required dermatomal blockage for the surgical field. For example we report laparoscopic Nissen fundoplication with ESPB from Th6, Th8, and Th10 levels and laparoscopic hysterectomy from Th9, Th10, and Th11 levels.

LA spread both cephalad and caudally is more extensive in ESPB when compared to other peri-paravertebral blocks [12]. We therefore advise that operators use the most sonographically visible point for block application, taking into account and keeping within the dermatomal coverage area.

Block success and effective analgesia are dependent on many factors and we are not able to explain the exact mechanism and sensorial coverage in ESPB. While there are differences in the spread of the LA in thoracic and lumbar vertebral applications, there are also differences reported within thoracic applications [14]. Although a mini review by De Cassai et al. [17] reported that a volume of 3.4 mL per segment was adequate, a cadaver study reported that 20 mL of LA spread between three to seven levels, averaging blockage of 4.6 levels [13]. On the other hand, some studies have reported large dermatomal spread with small volumes [18,19]. However these reports do not hold enough evidential merit to be generalized [15,20].

We took into account the dermatomal coverage of the surgical field in determining the volume and generally applied 20-30 mL LA in thoracic and 30-40 mL in lumbar areas. When we compared applied volume and block success, we were unable to determine any causative relationship. However, it is probable that high volume and concentration increase the success rate of ESPB.

Understanding the anatomy of the fascia between the erector spinae muscle and the transverse process may be the key to increasing block success. When applying LA in ESPB to the interfascial plane, presuming that there are two overlapping layers in which LA is applied between may lead to increased failure. We must consider that the deeper fascia that ESPB targets is multi-structured and that increased spread between these structures will lead to better block success and coverage [21]. We used the out-of-plane technique for ESPB in an overwhelming majority of our patients. We first made contact with the transverse process and applied the LA between these multiple layers. We hypothesize that this increased our block success rate although we do not have enough data to prove this.

Ultrasound-guided ESPB is a new and popular block technique and only two complications have been reported. One of these was pneumothorax and the second, from our institute, was motor weakness when ESPB was performed from a lower thoracic level [8,9]. Pneumothorax following ESPB is not expected when it is performed under ultrasound guidance but may be the result of loss of hand-eye coordination or miscalculating depth. Motor weakness may occur when the LA spreads to the lumbar plexus when performed from the lower thoracic or lumbar areas. We previously reported that lumbar ESPB leads to effects similar to lumbar plexus block and also demonstrated this spread radiologically [2,22]. Our report of ESPB from L4 being used for effective postoperative analgesia in hip, femur, and knee surgery is of clinical significance. Further studies are required to determine the relationship between volume and the LA spread, if one exists.

Local anaesthetic systemic toxicity (LAST) is typically manifested as central nervous system (CNS) toxicity (tinnitus, disorientation, and ultimately, seizures) or cardiovascular toxicity (hypotension, dysrhythmias, and cardiac arrest). The dose capable of causing CNS symptoms is typically lower than the dose and concentration result in cardiovascular toxicity. This is because the CNS is more susceptible to local anaesthetic toxicity than the cardiovascular system [23,24]. We observed LAST-related findings in three patients. Major CNS findings were aphasia/apathia in one patient, short-lasting loss of consciousness in another, while minor symptoms were vertigo/tinnitus in one patient. We observed no cardiovascular system findings.

The classical teaching that vascular absorption of LAs is highest with intercostal blocks followed by epidural and brachial plexus injections corresponds to clinical data demonstrating that the highest incidence of LAST occurs with paravertebral blocks, followed by upper extremity and trunk/lower extremity blocks [25]. The risk of LAST in interfascial plane blocks is generally the use of high volume and the spread of LA from the interfascial plane to the vascular-rich muscles and thereon to the systemic circulation. The time from interfascial block to peak plasma concentration of the LA is 30 minutes or more [24]. It is our opinion that LAST after ESPB is caused due to the spread of the LA to paravertebral and intercostal spaces and fast dissipation of the LA into the systemic circulation due to highly vascular muscle tissue surrounding the area of application.

None of the patients with complications had a history of spinal surgery. The LA in two consisted of 0.25% marcaine-0.5% lidocaine, one consisted of bilaterally applied 20 mL / 20 mL of 0.25% bupivacaine. It is noteworthy that no complications were observed in the group of patients in whom 60 mL of 0.375% marcaine was applied, yet complications occurred at lower concentrations of the LA. In our experience, concentrations of the LA did not exceed 2.5 mg/kg for bupivacaine and 4 mg/kg for lidocaine.

The rate of LAST in our series is 1.6%, which we consider to be relatively high. However, we did not observe any major LAST complications such as seizures or cardiac arrest. Complications defined herein are suspicious cases of minor LAST complications which may be due to the volume/concentration of the LA used. Literature reports very few cases of LAST after interfascial plane blocks. We believe that this complication is underreported as the symptoms may be mild (perioral numbness, tinnitus, agitation) and masked as sedation precedes the induction of general anaesthesia that immediately follows awake intubation or as the block is performed under anaesthesia. Larger case series and meta-analysis are required to determine the exact rate of LAST in ESPB.

Another analysis in this study was the rate of “block failure/lack of efficiency” and “inadequate spread for surgical procedure”. We must firstly clarify these definitions. Unlike peripheral nerve blocks it is impossible to directly determine block failure in interfascial blocks. For example, the radial nerve is either blocked or not blocked following brachial plexus block from the axillary region; therefore, the patient either has pain in the area innervated by the radial nerve or not. However, the evaluation of interfascial blocks includes many components and evaluation of the dermatomal spread is generally not adequate for the determination of block success [26,27]. While ESPB may only show effect in the paraspinal area, it may also lead to the blocking of the lateral aspect of the thoracoabdominal area and the mid-abdomen. This is may be due to several factors. First, while only the dorsal ramus of the thoracic nerves may be affected, in many cases the LA spreads anteriorly to the paravertebral space also affecting the ventral ramus. Sometimes, the LA may spread not anteriorly but laterally to the deeper fascia of the rhomboid muscle blocking off the lateral branches of the intercostal nerves and resulting in sensorial block of the mid abdomen and parasternal area [11,13,28]. The question of whether ESPB is effective on visceral pain as it is on somatic pain is still to be answered [3,29,30].

How can the success of ESPB be evaluated when the mechanism of action is yet to be understood? Dermatomal spread is far from being the answer. Can we say that sensorial/somatic blockage of the surgical area leads to effective analgesia? Or can we expect ESPB to lead to a complete block of a hemithorax or hemiabdomen [28]?

We determined to also use lack of efficiency as a definition apart from block failure. Therefore, our definition of block failure/lack of efficiency was NRS >6 in the first hour or requirement of rescue analgesia when ESPB is used as a component of multimodal analgesia. We observed that 12 patients had block failure/lack of efficiency (6.5%). These patients underwent varying procedures from shoulder to hip surgery and therefore had ESPB performed for differing indications. Further studies are required to analyze block failure/lack of efficiency as well as sensorial spread not only of the surgical field but of the back, side, and anterior.

We determined that 10 patients (5.5%) had inadequate spread for surgical procedure. In these, two patients undergoing mastectomy and axillary dissection had mild pain in the axilla. When considering that the sensorial innervation of the axilla comes from branches of the cervical plexus, this may be expected. In seven patients undergoing laparoscopic Nissen fundoplication, one had both moderate (NRS <6) left shoulder and mid-abdominal pain, which was accepted as inadequate spread for surgical procedure. It is noteworthy that while eight of these patients had “inadequate spread for surgical procedure” none required rescue analgesia.

Our study has several limitations. Firstly, the study was designed as a single-center, retrospective chart review. Therefore some block failures, etc. may not have been recorded or may have been overlooked. Another limitation is that sensorial evaluation of patients undergoing ESPB was not routinely performed. Also, nearly all ESPB were performed under general anaesthesia or sedoanalgesia meaning that some neurological findings of LAST may not have been observed.

## Conclusions

Our experience has demonstrated that when used as part of a multimodal analgesia plan, ESPB is an effective and safely performed interfascial plane block with a large range of indications. More experience must be reported to better understand the complication rates, mechanism of action, and factors that affect block failure/lack of efficiency.
